# Mental Health and Aging: Identifying Risk and Protective Factors of Anxiety and Depression in Older Women

**DOI:** 10.1002/agm2.70046

**Published:** 2025-10-25

**Authors:** Guilherme Augusto Santos Bueno, Renato Canevari Dutra da Silva, Elton Brás Camargo Júnior, Stephany Kindorly de Oliveira Bueno, Anabela Correia Martins, Ruth Losada de Menezes

**Affiliations:** ^1^ Postgraduate Program in Health Sciences and Technologies University of Brasília Brasília Brazil; ^2^ Department of Medicine University of Rio Verde Formosa Goiás Brazil; ^3^ Department of Odontology University of Rio Verde Rio Verde Goiás Brazil; ^4^ Department of Postgraduate University of Rio Verde Rio Verde Goiás Brazil; ^5^ Department of Physiotherapy Strategic Health Management Institute of the Federal District Brasília Brazil; ^6^ LabinSaúde Coimbra Health School Polytechnic of Coimbra, (ESTeSC‐IPC) Coimbra Portugal; ^7^ Postgraduate Program in Health Sciences Federal University of Goiás Goiânia Brazil

**Keywords:** anxiety, depression, postural balance, reaction time, seniors

## Abstract

**Objective:**

To identify risk and protective factors associated with anxiety and depression symptoms in older adult women, considering clinical, functional, and electrophysiological parameters.

**Methods:**

This cross‐sectional observational study involved 91 women aged 65 or older. Assessments included EEG‐derived valence and excitation levels, motor reaction time, body composition, and functional performance. Anxiety and depression were screened using the mind over mood anxiety inventory and the geriatric depression scale, respectively.

**Results:**

Anxiety symptoms were present in 28.6% of participants and depressive symptoms in 27.5%. Risk factors for anxiety included fear of falling (OR = 2.023, 95% CI = 1.554–2.944, *p* = 0.007), presence of depressive symptoms (OR = 2.254, 95% CI = 1.983–3.085, *p* = 0.008), and body fat percentage (OR = 1.543, 95% CI = 1.253–3.111, *p* = 0.011). Protective factors included valence (OR = 0.311, 95% CI = 0.272–0.544, *p* = 0.003), gait speed (OR = 0.674, 95% CI = 0.482–0.782, *p* = 0.023), and maximal expiratory pressure (OR = 0.755, 95% CI = 0.693–0.823, *p* = 0.027). For depressive symptoms, risk factors included fear of falling (OR = 1.983, 95% CI = 1.865*–*3.801, *p* = 0.023) and anxiety symptoms (OR = 1.765, 95% CI = 1.563–1.983, *p* = 0.008), while protective factors were cortical excitation (OR = 0.523, 95% CI = 0.425–0.693, *p* = 0.023) and expiratory pressure (OR = 0.635, 95% CI = 0.491–0.723, *p* = 0.021).

**Conclusion:**

Functional and emotional parameters, particularly valence and gait performance, suggest a potential protective role against anxiety and depression in aging women. The findings reinforce the importance of integrated assessments to support early detection and guide intervention planning, while further studies are recommended to strengthen these observations.

## Graphical abstract

Older women with increased fat percentage, depressive symptoms, and slower motor reaction times are at higher risk for anxiety and depression. In contrast, valence, muscle strength, gait speed, and respiratory function serve as protective factors. These findings highlight key intervention targets to promote mental health and functional well‐being in aging.

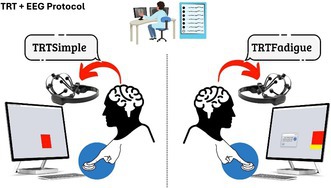

## 1 Introduction

In recent years, the population of older adults has increased significantly, especially in developing countries. This growth is primarily attributed to improvements in basic healthcare, physical and mental conditioning, and advances in disease prevention and treatment strategies [1, 2, 3]. Healthy aging is defined by three key components: a low probability of disease, the absence of cognitive or physical‐functional impairment, and active engagement in life [4, 5].

Mental health disorders are among the leading contributors to increased disability and decreased quality of life in older adults, largely due to the progressive decline in physical, cognitive, and psychosocial abilities [6, 7, 8, 9, 10]. These limitations go beyond the musculoskeletal system, affecting neurosensory‐motor integration, audiovisual processing, and the perception of temporal order [7, 9, 11]. Older adults often experience difficulties with object recognition, spatial orientation, and motor responses, all of which contribute to an elevated risk of falls [12].

Reaction time in response to balance loss is a critical component of fall prevention [13] requiring adequate neuromuscular coordination, muscle strength, and motor responsiveness [14]. Postural control is essential for maintaining balance, as it ensures the body's center of mass remains within the limits of stability without altering the base of support [15]. As aging is associated with a longer duration needed to execute postural adjustments, the risk of falling increases, further compromising functional capacity [16].

Among the cognitive and emotional disorders that affect the aging population, depression is particularly noteworthy [17, 18, 19]. It is a multifactorial condition that influences mood and affective regulation, with a substantial impact on functioning. Depression is shaped by biological, psychological, and social factors, and its primary symptoms include persistent sadness and a marked loss of interest or pleasure in nearly all daily activities [20, 21].

From an evolutionary perspective, anxiety is a natural and adaptive emotional response to perceived threats. When proportional to the situation, it can be recognized, managed, and even beneficial. However, distinguishing adaptive anxiety from pathological anxiety requires careful clinical evaluation [22]. Pathological anxiety is marked by persistent, excessive, and uncontrollable worry about future events, leading to heightened arousal and vigilance, often without an identifiable cause. This may result in defensive or avoidant behaviors [23].

Pathological anxiety is the most prevalent mental health disorder worldwide, affecting approximately 40 million adults annually [24, 25], Women are particularly vulnerable, not only to the emotional effects of anxiety but also to associated neuromotor impairments, which tend to be more pronounced compared to men [26]. Nearly a decade ago, Kertzman et al. [27] demonstrated that anxiety and depression contribute to anticipatory declines in functional ability, manifesting as slower reaction times and reduced motor and psychomotor speed.

Given these considerations, the present study aims to identify risk and protective factors associated with anxiety and depression symptoms in older adult women. The analysis includes personal characteristics, health status, body composition, motor reaction time, cortical excitation and valence, and overall functional performance.

## 2 Methods

### 2.1 Design and Ethical Aspects

This cross‐sectional, analytical observational study was approved by the Research Ethics Committee of the Evangelical University of Goiás (approval number: 3.738.640) and conducted in accordance with the principles of the Declaration of Helsinki. The study adhered to the methodological guidelines outlined in the Strengthening the Reporting of Observational Studies in Epidemiology (STROBE) statement [28]. All participants provided informed consent and signed a written informed consent form prior to enrollment.

### 2.2 Sample

A pilot study involving 10 volunteers was initially conducted to estimate the appropriate sample size. The sample size calculation was performed using G*Power software (version 3.1.9.2; Franz Faul, Universität Kiel, Germany) [29], based on intergroup variance in Simple Motor Reaction Time (MRT) related to the presence of anxiety and depressive symptoms. The minimum required sample to detect a clinically relevant and statistically significant difference was *N* = 84, assuming an effect size (*r*) = 1.32, *p* < *0.05*, and statistical power of 0.95.

After recruitment, screening, and completion of evaluations, the final sample consisted of 91 older adult women aged 65 years or older. Inclusion criteria were as follows: (i) ability to stand and walk independently; (ii) good general health and eutrophic status; (iii) literate or illiterate; (iv) voluntary participation with signed informed consent; (v) body mass index (BMI) < 30 kg/m^2^; and (vi) preserved cognitive function, defined as a Mini‐Mental State Examination (MMSE) score > 18 points [30].

Exclusion criteria included: (i) neurological diseases and/or sequelae; (ii) vestibular disorders; (iii) uncorrected visual impairments; (iv) orthopedic conditions such as amputations, recent fractures, or a history of ankle sprain within the past 6 months; (v) alcohol consumption within 24 h prior to assessment; (vi) self‐reported spinal osteoarthritis and/or the presence of lower limb endoprosthesis; and (vii) a medical diagnosis of rheumatoid arthritis.

To ensure the internal validity of neurophysiological and functional measurements, strict exclusion criteria were applied to control for potential confounding variables such as neurological, vestibular, musculoskeletal, and cardiopulmonary disorders. These conditions could introduce artifacts or bias in EEG recordings, motor reaction time, and physical performance. Although these criteria may have excluded individuals with multimorbid profiles common in older populations, they enabled a more precise examination of the specific predictors of anxiety and depression. While this design improves the internal consistency of the findings, it may limit external generalizability to frailer or more clinically complex older adults.

Participants were community‐dwelling older women recruited from public health centers located in a mixed urban–rural region of Central Brazil. Educational backgrounds varied: 34.1% had no formal education, 45.1% had incomplete or complete elementary education, and 20.8% had completed secondary or higher education. Most participants belonged to low‐income households, reflecting the socioeconomic profile typically served by the Brazilian Unified Health System (*Sistema Único de Saúde* [SUS]). While the sample is representative of a socially vulnerable segment of older adult women in Latin America, findings should be interpreted cautiously when generalizing to higher‐income or male populations, considering the known gender and socioeconomic influences on emotional health and functional capacity in aging.

### 2.3 Assessment Instruments for Anxiety and Depressive Symptoms

Depressive symptoms were assessed using the Geriatric Depression Scale (GDS), a widely validated and reliable instrument for evaluating depressive disorders in older adults. The scale comprises 30 dichotomous questions with “yes” or “no” response options [31]. The items are designed for ease of comprehension, with limited variability in response interpretation. The cutoff scores used were: 0–10 (no depression), 11–20 (moderate depression), and 21–30 (severe depression) [32].

Anxiety symptoms were evaluated using the *Mind Over Mood Anxiety Inventory*, a self‐administered questionnaire consisting of 24 items, each addressing a distinct symptom of anxiety. Participants rated the frequency of each symptom over the past week using a 4‐point Likert scale ranging from 0 (“none of the time”) to 3 (“most of the time”) [33]. Based on the total score, participants were categorized as follows: < 19 (euthymic), 19–36 (mild anxiety), 37–54 (moderate anxiety), and 55–72 (severe anxiety) [34].

### 2.4 Instruments for Assessing Demographic Data, Health Aspects, and Functional Abilities

The study was conducted in two stages. In the first stage, participants provided written informed consent and completed an initial health screening. On a separate scheduled day, they underwent assessments of functional capacity, respiratory performance, motor reaction time, and completed questionnaires for identifying symptoms of depression and anxiety. Each session lasted approximately 40 min per participant.

During this initial stage, an interview was conducted to collect sociodemographic information, self‐rated physical health, subjective health perception, and fall history within the previous year.

Subsequently, cognitive screening was performed using the Mini‐Mental State Examination (MMSE), which evaluates orientation, memory, attention, and language skills. The instrument comprises two parts: the first assesses orientation, memory, and attention (maximum score: 21 points), and the second evaluates language and comprehension (maximum score: 9 points), for a total score of 30. Scores below 18 were considered indicative of cognitive impairment [30].

Physical activity levels were assessed using the International Physical Activity Questionnaire (IPAQ), adapted for older adults. The questionnaire estimates energy expenditure in MET‐minutes per week by multiplying the MET value for each activity intensity (light: 3.3 METs; moderate: 4.0 METs; vigorous: 8.0 METs) by the number of days and duration in minutes. Participants were classified as very active, active, irregularly active, or sedentary [35].

To evaluate fear of falling, the Falls Efficacy Scale–International (FES‐I Brazil) was applied. This 16‐item scale assesses concern about falling while performing common daily tasks. Each item is scored on a 4‐point scale (1–4), with total scores ranging from 16 (no concern) to 64 (extreme concern). Scores above 27 indicate a high fear of falling [36, 37].

### 2.5 Stage 2: Motor Reaction Time and EEG Assessment

The second stage began with the Motor Reaction Time (MRT) test, which included two conditions:

- *Simple MRT*: Participants responded as quickly as possible to a red square that appeared at random intervals (1.5–6.5 s) in the center of the screen by pressing the spacebar.
- *Fatigue MRT*: Participants followed a red bar moving horizontally across the screen. They were instructed to press and hold the spacebar as soon as the bar appeared and release it once it disappeared.

After a familiarization period (five runs), participants performed 10 trials of each condition [38]. Throughout the MRT tasks, EEG signals were recorded simultaneously to capture cortical activity associated with motor and attentional demands.

To record EEG signals, the study utilized the EMOTIV EPOC+ (Emotiv Inc., San Francisco, USA) [39, 40], a system validated for scientific research [41, 42, 43]. Electrode placement followed the International 10–20 system, ensuring coverage of the frontal, prefrontal, temporal, parietal, and occipital lobes [44]. A resting‐state EEG baseline was recorded with the following protocol:

- 3 s of preparation (countdown),
- 15 s with eyes open (countdown),
- 3 s of preparation (countdown),
- 15 s with eyes closed (countdown).

**FIGURE 1:**
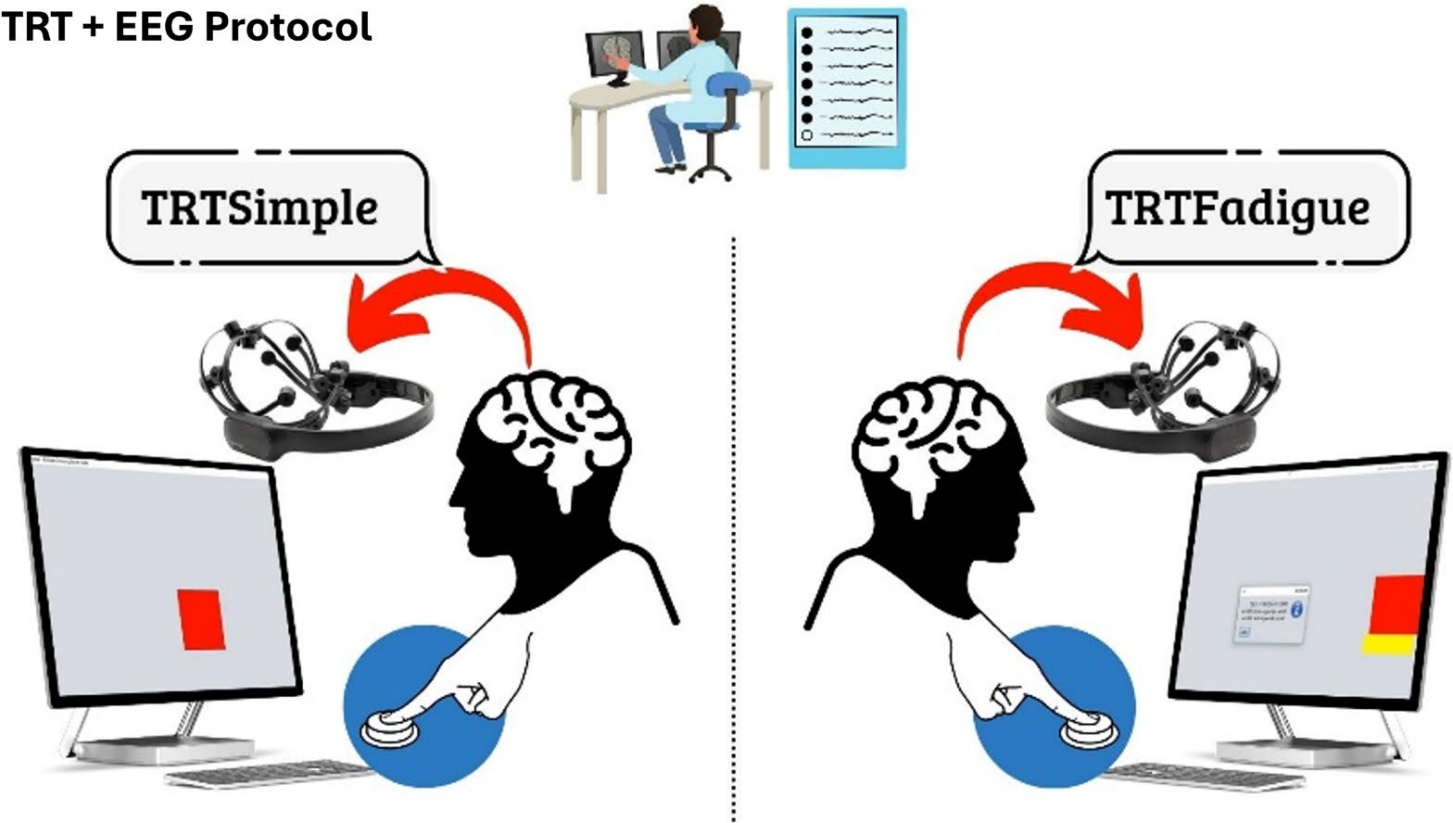
EEG acquisition protocol during motor reaction time tasks. *Source:* Authors' own elaboration.

A schematic of this protocol is shown in Figure 1.

### 2.6 Respiratory Function Assessment

Respiratory capacity was assessed using *manovacuometry* to measure maximal inspiratory pressure (MIP) and maximal expiratory pressure (MEP). Participants were seated with knees flexed at 90°, and elbows supported on their thighs. Three trials were conducted for each measure, with 1‐min rest intervals, and the highest values were recorded [45]. Reference values for women were: MIP = 26–73 cmH_2_O; MEP = 40–140 cmH_2_O [46].

Peak expiratory flow (PEF) was evaluated using a peak flow meter. Following a full inspiration, participants executed three maximum‐effort expirations, again with 1‐min rest intervals. The highest value among trials was used [47].

### 2.7 Functional and Mobility Assessments

Balance was evaluated using the 4‐Stage Balance Test. Participants were asked to maintain each of four progressively difficult positions for 10 s, without external support: (1) feet side by side; (2) semi‐tandem stance; (3) full tandem stance; (4) single‐leg stance [48].

Functional mobility was measured using the Timed Up‐and‐Go (TUG) test. Participants stood up from a chair, walked 3 m, turned, returned, and sat back down. Timing began at the “go” command and ended once the participant was seated. A time ≤ 10 s was considered indicative of preserved mobility [49, 50].

Gait speed was assessed using the 10‐Meter Walk Test. Participants walked a 20‐m corridor at their fastest safe pace, with or without assistive devices [51]. This test has demonstrated high reliability in adults (coefficient ≥ 0.903) [52]. The Gait Analyzer mobile app was used alongside this test. The app utilizes smartphone‐based sensors (tri‐axial accelerometer, gyroscope, and magnetometer, if available) to compute real‐time gait parameters, including velocity, step time, step length, cadence, and symmetry [53, 54].

The 30‐second chair stand test (30s STS) was performed to evaluate lower limb strength and fall risk. Participants were instructed to sit on a chair, place their hands on opposite shoulders (crossing their wrists), position their feet hip‐width apart, and keep their back straight. Upon hearing “GO”, they repeatedly stood up and sat down for 30 s. The total number of completed repetitions was recorded as [55]. The 30s STS test is considered a reliable indicator of fall risk in community‐dwelling older adults [56, 57].

Finally, handgrip strength was assessed using a Jamar dynamometer, which is widely recognized as the gold standard for handgrip strength measurement. Participants were seated comfortably with their shoulders slightly adducted, elbows flexed at 90°, and forearms in a neutral position. Wrist positioning varied between 0° and 30° of extension. Three measurements were taken on each upper limb, with 1‐min rest intervals between trials, and the highest value was recorded [58]. Several studies have reported high reliability and validity for this type of dynamometer, reinforcing its use in scientific and clinical settings [59, 60].

**FIGURE 2:**
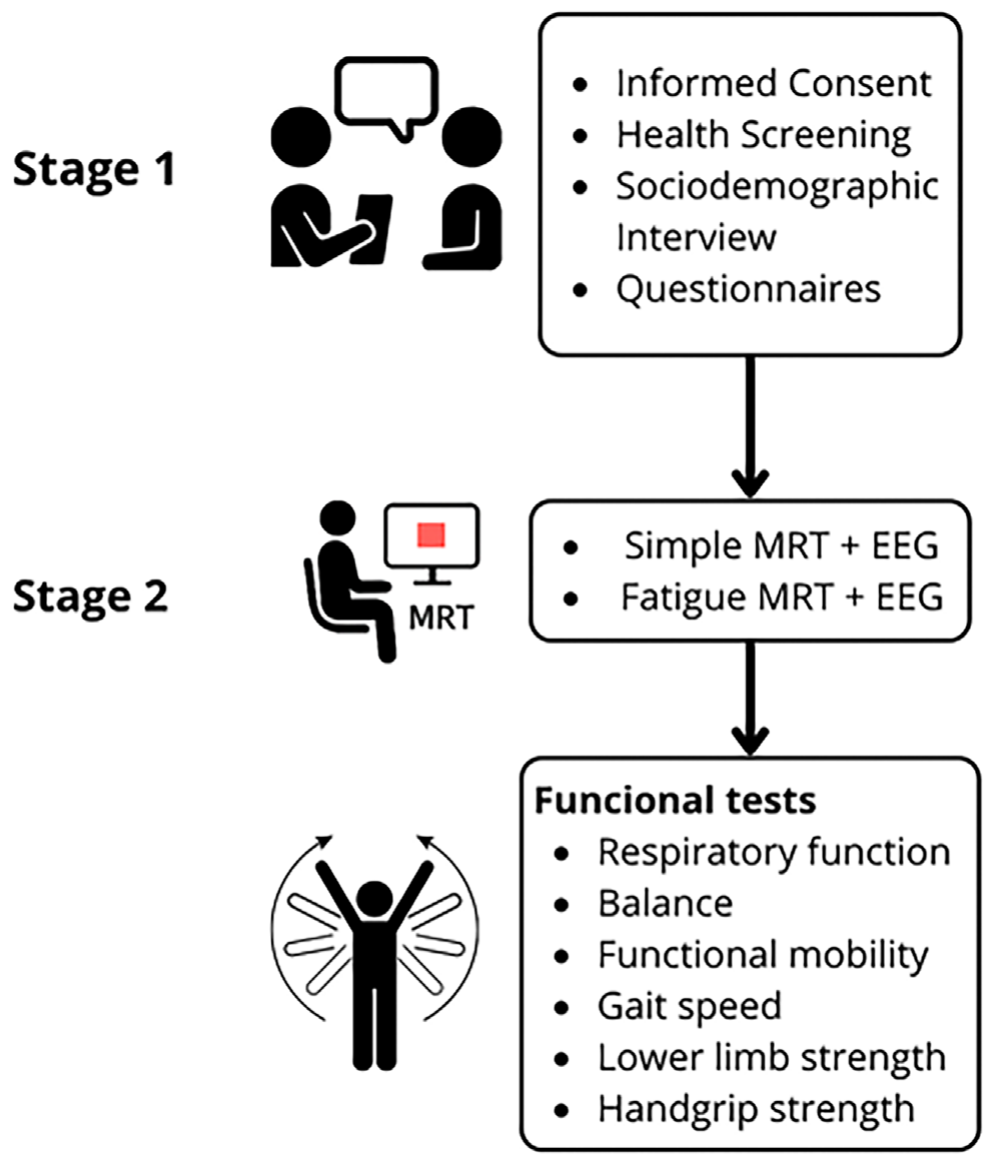
Evaluation protocol and timeline of procedures across stages 1 and 2. *Source:* Authors' own elaboration.

A detailed schematic of the full evaluation protocol is presented in Figure 2, illustrating the timeline and structure of procedures implemented across the two assessment stages. In stage 1, participants underwent consent, health screening, and a structured sociodemographic interview, followed by the administration of validated questionnaires addressing cognitive status, physical activity, depressive and anxiety symptoms, and fear of falling. This stage also included measurements of respiratory function, balance, mobility, strength, and gait performance. In stage 2, participants completed both simple and fatigue motor reaction time (MRT) tasks while electroencephalographic (EEG) activity was simultaneously recorded. This diagram visually summarizes the sequencing and integration of multidimensional assessments adopted in the present study.

### 2.8 Datal Analysis

To extract meaningful features from the EEG data, pre‐processing was applied to eliminate noise and irrelevant information. EEG data were analyzed offline using the EEGLAB toolbox [61] in *Matlab R2019b* (The Mathworks, Natick, MA, USA). The pre‐processing steps included: 1. Downsampling the signal to 250 Hz; 2. Applying a 0.01–45 Hz Butterworth filter; 3. Performing independent component analysis (ICA) to remove artifacts [62, 63].

Artifact‐containing components were manually identified for each subject based on their energy spectra, visual patterns, and spatial energy distribution over the cortex. These components were discarded before performing the inverse ICA. After artifact rejection, the datasets were filtered into the following traditional EEG frequency bands: Delta (*δ*): 0.5–4 Hz; Theta (*θ*): 4–8 Hz; Alpha (*α*): 8–13 Hz; Beta (*β*): 13–30 Hz; Gamma (*γ*): 30–45 Hz [64].

Based on a person's EEG signal, the level of arousal was determined by calculating the ratio of beta (12–28 Hz) and alpha (8–12 Hz) brain waves. The EEG signal was measured at four sites (i.e., electrodes) in the prefrontal cortex: AF3, AF4, F3, and F4. Beta *β* waves are associated with an alert or excited mental state, whereas alpha *α* waves are more dominant in a relaxed state. Alpha activity has also been linked to brain inactivation. Thus, the beta/alpha ratio is a reasonable indicator of a person's state of arousal [65]. Specifically, the level of excitement was computed as follows:

$$\begin{array}{c} \text{Excitation level}\\ \;=\left(\text{ΒF3}+\text{ΒF4}+\text{ΒAF3}+\text{ΒAF4}\right)/\left(\alpha \text{F3}+\alpha \text{F4}+\text{αAF3}+\alpha \text{AF4}\right) \end{array}$$

To determine the valence level, the activation levels of the two cortical hemispheres were compared. A large number of EEG studies [66, 67] have shown that the left frontal area is associated with more positive affects and memories, and the right hemisphere is more involved in negative emotions. Thus, F3 and F4 were determined as reference electrodes, as they are the most used positions to look at this alpha/beta activity related to valence, as they are located in the prefrontal lobe, which plays a crucial role in emotional regulation and experience conscious. The valence values were computed by comparing the alpha power *α* in the F3 and F4 channels [65]. Concretely, the valence level was computed as follows:

$$\text{Valence}=\left(\alpha \text{F4}/\beta \text{F4}\right)-\left(\alpha \text{F3}/\beta \text{F3}\right)$$

All statistical analyses were performed using IBM SPSS Statistics, version 26.0 (IBM Corp., Armonk, NY, USA). Descriptive statistics were computed for all variables and expressed as means and standard deviations for continuous variables, and as frequencies and percentages for categorical variables.

Normality was assessed using the Shapiro–Wilk test. Group comparisons between participants with and without anxiety or depression symptoms were conducted using independent‐samples *t*‐tests for normally distributed data and Mann–Whitney *U* tests for non‐normal distributions. The chi‐square test or Fisher's exact test was used for categorical variables, as appropriate.

Binary logistic regression models were applied to identify independent predictors of anxiety and depression symptoms. First, univariate logistic regressions were conducted for each variable. Those with *p* < 0.10 were included in multivariate models using a backward stepwise elimination procedure. All models were adjusted for age and MMSE score. The results were presented as odds ratios (OR) with 95% confidence intervals (CI), and statistical significance was set at *p* < 0.05. Model adequacy was tested using the Hosmer–Lemeshow goodness‐of‐fit test. Multicollinearity was evaluated using the variance inflation factor (VIF), with a threshold of 2.5. The area under the receiver operating characteristic (ROC) curve was also calculated to assess the discriminative capacity of the final models.

## 3 Results

The odds ratio analysis revealed that in older adult women over 60, several factors were identified as risk factors for anxiety. These included fear of falling (OR = 2.023, *p* = 0.007), depressive symptoms assessed using the Geriatric Depression Scale (OR = 2.254, *p* = 0.008), body fat composition (OR = 1.543, *p* = 0.011), simple motor reaction time (TRT_simple, ms) (OR = 1.684, *p* = 0.017), initial fatigue reaction time (TRT_initial fatigue, ms) (OR = 1.423, *p* = 0.020), and history of falls (OR = 2.505, *p* = 0.014). On the other hand, the findings indicated that valence and functional abilities, such as respiratory function, gait speed, and step length, acted as protective factors against anxiety (Table 1).

**TABLE 1:** Association between anxiety in older adult women and age, body composition, personal factors, history of falls, and functional abilities.

	Total (*n* = 59) average (SD) or *n* (%)	Anxiety (*n* = 36)	Control group (*n* = 23)	OR (95% CI)	*p*
		Average (SD) or *n* (%)	Average (DP) or *n* (%)		
Age (years)	71.31 (8.09)	74.24 (7.92)	70.36 (5.70)	1.000 (1.000–1.011)	0.003^a^
*Body composition*					
BMI (kg/m^2^)	26.07 (4.11)	25.30 (3.80)	27.37 (4.39)	1.012 (0.995–1.034)	0.102^a^
Muscle (%)	25.88 (2.68)	22.81 (2.98)	26.00 (2.14)	0.583 (0.322–0.683)	0.032^a^
Fat (%)	37.54 (6.20)	39.97 (6.28)	31.50 (6.08)	1.543 (1.253–3.111)	0.011^a^
Visceral fat (%)	9.22 (2.55)	8.97 (2.61)	9.64 (2.44)	0.951 (0.900–1.012)	0.106^a^
*Personal factors*					
MMSE (score)	24.95 (4.36)	24.62 (5.24)	25.50 (2.20)	1.012 (0.994–1.045)	0.075^a^
FES‐I BRASIL (score)	31.39 (9.62)	34.86 (9.31)	25.55 (7.07)	2.023 (1.554–2.944)	0.007^a^
GDS (score)	15.29 (3.92)	18.65 (3.31)	13.00 (3.85)	2.254 (1.983–3.085)	0.008^a^
*History of falls*					0.014^b^
Yes	35 (59.3)	25 (42.4)	10 (16.9)	2.505 (1.201–5.200)	
No	24 (40.7)	12 (20.3)	12 (20.3)	1	
*Neuromechanical assessment*					
MRT_SIMPLE (ms)	836.69 (158.50)	932.95 (169.46)	674.82 (198.76)	1.684 (1.253–1.984)	0.017^a^
MRT_INITIAL FATIGUE (ms)	1120.80 (198.86)	1218.32 (102.85)	956.77 (157.09)	1.423 (1.132–1.623)	0.020^a^
MRT_FINAL FATIGUE (ms)	958.10 (189.88)	904.89 (116.22)	1047.59 (138.87)	1.004 (1.001–1.000)	0.218^a^
Cortical excitation (%)	0.98 (0.09)	0.98 (0.10)	0.98 (0.08)	1.011 (0.994–1.032)	0.102^a^
Valence (%)	0.94 (0.12)	0.90 (0.06)	0.97 (0.05)	0.311 (0.272–0.544)	0.003^a^
*Functional abilities*					
P_IP_ (cmH_2_O)	85.61 (10.77)	75.50 (13.44)	89.09 (5.03)	0.823 (0.711–0.875)	0.042^a^
P_EP_ (cmH_2_O)	96.13 (10.12)	82.69 (9.83)	98.41 (9.56)	0.755 (0.693–0.823)	0.027^a^
Peak Flow (l/min)	243.22 (63.34)	234.05 (73.88)	258.64 (36.42)	1.000 (1.000–1.000)	0.127^a^
Handgrip strength (kg/f)	21.76 (3.70)	21.24 (3.39)	22.64 (4.10)	0.983 (0.954–1.000)	0.092^a^
30‐s Sit‐to‐stand (score)	9.10 (3.42)	9.00 (3.96)	9.23 (2.31)	0.953 (0.905–1.000)	0.083^a^
Timed up‐and‐go (s)	9.95 (2.88)	10.68 (3.19)	7.73 (1.72)	0.932 (0.884–0.984)	0.013^a^
4‐Stage balance test (score)	3.08 (0.93)	2.28 (0.99)	3.56 (0.84)	0.685 (0.613–0.855)	0.039
Gait speed (m/s)	1.54 (0.50)	1.21 (0.49)	1.53 (0.50)	0.674 (0.482–0.782)	0.023^a^
Step length (m)	0.20 (0.41)	0.24 (0.43)	0.14 (0.35)	0.335 (0.094–0.522)	0.012^a^
Step time (s)	0.73 (0.45)	0.68 (0.47)	0.82 (0.39)	0.722 (0.394–1.320)	0.288^a^
Cadence (steps/min)	110.97 (14.37)	111.54 (15.53)	110.00 (12.45)	0.812 (0.652–0.932)	0.062^a^

Abbreviations: BMI, Body Mass Index; FES‐I, Falls Efficacy Scale‐International; GDS, Geriatric Depression Scale; kg, kilogram; kg/m^2^, kilogram/square meter; m, meters; m/s, meter/s; MMSE, Mini Mental State Examination; MRT, motor reaction time; *n*, sample; P_EP_, peak expiratory pressure; P_IP_, peak inspiratory pressure; SD, standard deviation.

^a^ Student's *t*‐test.

^b^ Chi‐square.

Body composition and functional attributes were identified as protective factors against depression. Specifically, muscle percentage (OR = 0.493, *p* = 0.021), cortical excitation (OR = 0.523, *p* = 0.023), valence (OR = 0.195, *p* < 0.001), gait speed (OR = 0.325, *p* = 0.008), physical performance (OR = 0.681, *p* = 0.043), step length (OR = 0.334, *p* = 0.019), and expiratory pressure (OR = 0.635, *p* = 0.021) were all associated with a lower likelihood of depression (Table 2).

**TABLE 2:** Association between depressive symptoms in older adult women and age, body composition, personal factors, history of falls, and functional abilities.

	Total (*n* = 54) average (SD) or *n* (%)	Depressive symptoms (*n* = 31)	Control group (*n* = 23)	OR (95% CI)	*p*
		Average (SD) or *n* (%)	Average (DP) or *n* (%)		
Age (years)	71.31 (8.09)	71.31 (6.18)	71.30 (10.03)	0.995 (0.995–1.000)	0.518^a^
*Body composition*					
BMI (kg/m^2^)	26.07 (4.11)	26.32 (3.81)	25.78 (4.50)	0.992 (0.973–1.012)	0.472^a^
Muscle (%)	25.88 (2.68)	21.62 (2.61)	26.19 (2.77)	0.493 (0.375–0.623)	0.021^a^
Fat (%)	37.54 (6.20)	38.19 (5.69)	31.78 (6.78)	1.642 (1.382–3.124)	0.034^a^
Visceral fat (%)	9.22 (2.55)	9.59 (2.45)	7.78 (2.64)	1.574 (1.422–2.952)	0.042^a^
*Personal factors*					
MMSE (score)	24.95 (4.36)	25.37 (3.82)	24.44 (4.94)	0.995 (0.974–1.212)	0.435^a^
FES‐I BRASIL (score)	31.39 (9.62)	36.66 (7.79)	25.15 (7.70)	1.983 (1.865–3.801)	0.023^a^
Anxiety inventory (score)	24.00 (3.51)	29.84 (2.21)	17.07 (1.85)	1.765 (1.563–1.983)	0.008^a^
*History of falls*					0.021^b^
Yes	35 (59.3)	19 (32.2)	16 (27.1)	1.423 (1.322–2.400)	
No	24 (40.7)	13 (22.0)	11 (18.6)	1	
*Neuromechanical assessment*					
MRT_SIMPLE (ms)	836.69 (158.50)	994.84 (181.14)	767.78 (128.76)	1.895 (1.691–3.251)	0.029^a^
MRT_INITIAL FATIGUE (ms)	1120.80 (198.86)	1225.94 (182.41)	896.19 (197.95)	2.213 (1.853–5.232)	0.009^a^
MRT_FINAL FATIGUE (ms)	958.10 (189.88)	990.78 (186.19)	808.63 (102.69)	1.452 (1.313–2.453)	0.035^a^
Cortical excitation (%)	0.96 (0.28)	0.93 (0.14)	0.98 (0.08)	0.523 (0.425–0.693)	0.023^a^
Valence (%)	0.84 (0.39)	0.72 (0.13)	0.97 (0.05)	0.195 (0.124–0.242)	< 0.001^a^
*Functional abilities*					
P_IP_ (cmH_2_O)	85.61 (10.77)	990.78 (186.19)	808.63 (102.69)	1.453 (1.312–2.454)	0.035^a^
P_EP_ (cmH_2_O)	96.13 (10.12)	69.25 (2.27)	87.52 (12.26)	0.635 (0.491–0.723)	0.021^a^
Peak flow (l/min)	243.22 (63.34)	89.25 (6.53)	91.11 (11.55)	1.023 (0.995–1.125)	0.147^a^
Handgrip strength (kg/f)	21.76 (3.70)	234.69 (70.34)	253.33 (53.42)	1.002 (0.995–1.000)	0.729^a^
30‐s sit‐to‐stand (score)	9.10 (3.42)	7.09 (2.91)	10.30 (3.64)	0.681 (0.351–0.853)	0.043^a^
Timed up‐and‐go (s)	9.95 (2.88)	10.38 (2.54)	8.44 (3.21)	1.571 (1.352–2.264)	0.035^a^
4‐Stage balance test (score)	3.08 (0.93)	3.16 (0.95)	3.00 (0.92)	0.932 (0.793–1.091)	0.420^a^
Gait speed (m/s)	1.54 (0.50)	1.10 (0.50)	1.48 (0.51)	0.325 (0.234–0.491)	0.008^a^
Step length (m)	0.20 (0.41)	0.15 (0.06)	0.21 (0.08)	0.334 (0.094–0.433)	0.019^a^
Step time (s)	0.73 (0.45)	0.75 (0.44)	0.70 (0.47)	0.793 (0.431–1.442)	0.447^a^
Cadence (steps/min)	110.97 (14.37)	107.25 (12.41)	115.37 (15.47)	0.992 (0.996–1.000)	0.713^a^

Abbreviations: BMI, Body Mass Index; FES‐I, Falls Efficacy Scale‐International; GDS, Geriatric Depression Scale; kg, kilogram; kg/m^2^, kilogram/square meter; m, meters; m/s, meter/s; MMSE, Mini Mental State Examination; MRT, motor reaction time; *n*, sample; P_EP_, peak expiratory pressure; P_IP_, peak inspiratory pressure; SD, standard deviation.

^a^ Student's *t*‐test.

^b^ Chi‐square.

## 4 Discussion

The present study aimed to identify risk and protective factors for anxiety and depression in older adult women, considering personal characteristics, health status, body composition, motor reaction time, cortical excitation, valence, and functional abilities. The final sample comprised 90 physically active women aged 65 years or older.

One of the key findings underscores the clinical and scientific relevance of body fat percentage as a significant marker for identifying physical and cognitive health risks in older adults. The results revealed that an increased body fat percentage was a significant risk factor for both anxiety and depression. This aligns with previous studies indicating that nutritional status strongly influences mental health and the development of mood disorders [68]. Obesity has been associated with higher prevalence rates of depression and anxiety, likely due to mechanisms involving neuroinflammation, hormonal dysregulation, and reduced levels of physical activity [69, 70, 71]. Anxiety further contributes to mood instability, diminished motivation for daily activities, and sedentary behavior, establishing a vicious cycle that exacerbates symptoms, increases fall risk, and worsens overall health outcomes.

From a neuroscientific perspective, valence emerged as a crucial indicator of neural activity, capturing emotional states directly associated with anxiety and depression. The absence of a statistically significant correlation between arousal and anxiety time should not be interpreted as a negative finding; rather, it reinforces valence as the most salient electrophysiological marker in these conditions. Valence reflects the balance between positive and negative emotional states and plays a key role in the regulation of mood disorders. The observed protective role of higher valence scores further supports the role of emotional regulation mechanisms in mitigating vulnerability to psychological distress.

Another pivotal finding of this study concerns the relationship between emotional health and functional performance. The data indicate that anxiety and depression are linked to longer motor reaction times, increased hesitation errors, and heightened cognitive demand for spatial processing—patterns consistent with prior research [72, 73, 74]. These outcomes suggest compromised cognitive efficiency, likely due to increased attentional resource allocation toward error monitoring and correction [75].

The association between fear of falling, functional mobility (as measured by the TUG test), and mental health also became evident. Depression and anxiety were strongly linked to increased fall risk, potentially explained by reduced balance, slower gait speed, postural instability, loss of independence, cognitive decline, and greater frailty. Furthermore, a prior history of falls was identified as a major risk factor for anxiety, indicating that the experience of falling may reinforce fear, diminish confidence in mobility, and intensify psychological distress [76]. These findings align with evidence suggesting that fear of falling is both a consequence and a predictor of mental health decline, perpetuating a cycle of reduced physical activity, impaired mobility, and emotional deterioration.

Age‐related declines in postural control, muscular strength, mobility, and respiratory function also contribute to these outcomes. Regardless of whether participants were classified as anxious or depressed, the natural aging process imposes functional limitations that may predispose individuals to anxiety, which can subsequently evolve into depression [17, 77]. This progression highlights the need for early intervention to delay functional decline and minimize the cumulative effects of psychological disorders.

Another essential observation concerns the role of anxiety in impairing sensory information processing and physical balance. Anxiety may disrupt sensory filtering and prioritization, increasing postural sway, decreasing balance control, and heightening fall risk [78, 79]. These effects are compounded by age‐related sensory processing delays, slower reflex responses, and impaired stimulus discrimination, all of which contribute to instability in older adults [80, 81].

Crucially, anxiety emerged as a primary antecedent to depression, with symptoms often worsening over time due to delayed diagnosis, physical and cognitive decline, and diminished quality of life. Nevertheless, the findings also point to actionable solutions. Targeted interventions—such as physical exercise, strength training, balance and mobility programs, flexibility routines, and regular health monitoring—have demonstrated efficacy in reducing anxiety and preventing its progression into depression [78, 82, 83]. Exercise‐based strategies have also been shown to improve reaction time, cognitive function, cardiorespiratory capacity, and overall quality of life, reinforcing their role as non‐pharmacological approaches for mood disorder mitigation [84]. By enhancing functional independence and emotional regulation, these approaches offer viable pathways to reduce the impact of depression and anxiety among older adults.

It is important to note that the study sample consisted exclusively of women, and the cross‐sectional design does not allow for causal inference. However, to the best of our knowledge, no prior studies have incorporated motor reaction time in examining risk factors for anxiety and depression in older adults.

## 5 Conclusion

By broadening the scope of investigation, this study offers strategic insights for designing future interventions aimed at older women affected by psychogenic disorders. Psychogenic conditions related to aging are often associated with alterations in executive functioning, attention, and information processing speed, alongside functional decline. However, this study highlights several protective variables that can be actively strengthened. Promoting physical fitness, muscular strength, balance, mobility, and emotional regulation may foster mental well‐being, preserve functional independence, and improve quality of life in older women. These findings underscore the importance of early detection and preventive strategies and lay the groundwork for future research and clinical programs aimed at mitigating the impact of anxiety and depression in aging populations, although further investigations are recommended to deepen and consolidate these obersavations.

## Author Contributions

G.A.S.B.: conceptualized the study, analyzed and interpreted the data, and wrote the manuscript. R.C.D.S., E.B.C.J., and S.K.O.B.: analyzed the data and critically revised the manuscript for important intellectual content. R.L.M. and A.C.M.: contributed to the study's concept and design, supervised the research, and critically revised the manuscript for significant intellectual content.

## Ethics Statement

This study was approved by the Research Ethics Committee of the Evangelical University of Goiás (CAAE: 18276419.7.0000.5076; Approval number: 3.738.640).

## Consent

All participants provided written informed consent.

## Conflicts of Interest

The authors declare no conflicts of interest.
